# The Temporal pathological changes of venous catheter-related fibrin sheath and their implications for nursing practice

**DOI:** 10.1371/journal.pone.0337478

**Published:** 2025-12-30

**Authors:** Wenshuai Tang, Limei Deng, Haimin Qin

**Affiliations:** 1 Nursing College, Guilin Medical University, Guilin, Guangxi, China; 2 The First Affiliated Hospital of Guilin Medical University, Guilin, Guangxi, China; 3 Guigang City People’s Hospital, Guigang, Guangxi, China; Baylor College of Medicine, UNITED STATES OF AMERICA

## Abstract

**Background:**

To investigate the time-sequential variation patterns of the impact of venous catheter-related fibrin sheath (FS) on blood vessels during the ultra-early period (12 hours to 4 days) after catheterization, and provide a basis for optimizing clinical catheter management strategies.

**Methods:**

A rabbit ear marginal vein indwelling catheter model (n = 40) was established and randomly divided into 12h, 1d, 2d, 3d, and 4d indwelling groups (8 rabbits per group). After sampling, Hematoxylin-Eosin **(**HE**)** staining was performed to systematically evaluate infusion patency, FS thickness, FS structural integrity, thrombus area, inflammatory cell infiltration, and venous edema. Welch’s ANOVA, trend tests, and Spearman correlation analysis were used for data analysis.

**Results:**

The patency of the catheters decreased significantly with increasing indwelling time (p < 0.001), with the rate dropping from 87.5% at 12 hours to 0% at 4 days, accompanied by a complete blockage rate of 75.0% at the latter time point. Concurrently, the thrombus area exhibited an extremely significant increase (p < 0.001), from 0.12 ± 0.03 mm² at 12h to 1.86 ± 0.20 mm² at 4 days (η² = 0.976). Regarding the fibrin sheath (FS), its thickness increased extremely significantly (p < 0.001) from 23.5 ± 4.2 μm at 12h to 156.7 ± 10.8 μm at 4 days (η² = 0.986). Furthermore, the integrity of the FS improved over time (p < 0.001), shifting from a state of predominantly incomplete continuity (87.5%) at 12h to predominantly complete continuity (87.5%) at 4 days.The inflammatory response intensified markedly, with the inflammatory cell count significantly increasing from 12.3 ± 2.5 cells/field at 12h to 85.4 ± 6.3 cells/field at 4 days (p < 0.001). The proportion of samples exhibiting severe inflammation rose from 0% to 62.5% (p < 0.001). Similarly, the degree of venous edema worsened significantly (p < 0.001), shifting from predominantly mild edema (62.5%) at 12h to severe edema (62.5%) at 4 days.Key time nodes were identified: 48 hours represented a critical point, characterized by a thrombus area >0.75 mm², FS thickness >78 μm, and a prevalence of moderate-to-severe inflammation and edema exceeding 75%. By 72 hours, a high-risk point was observed, marked by a complete blockage rate >50%, severe edema >25%, and FS complete rupture >50%.

**Conclusion:**

FS rapidly forms and dynamically evolves during the ultra-early period after catheterization, leading to time-dependent exacerbation of mechanical obstruction, thrombosis, and vascular inflammatory injury. The study identifies 48–72 hours as a critical intervention window, recommending enhanced clinical assessment and timely intervention (e.g., optimizing catheter replacement timing) to reduce the risk of catheter-related complications.

## Introduction

Fibrin sheath (FS) represents a defensive physiological response of the body to the foreign body of an implanted catheter [1–3]. When a venous catheter is inserted into a blood vessel, components such as proteins, platelets, and fibrinogen in the blood rapidly deposit on the catheter surface, triggering the coagulation cascade and gradually forming an FS structure that wraps around the catheter. This process is the body’s self-protection against foreign bodies, but it actually leads to numerous adverse consequences [4–7]. As a core infusion pathway for fluid resuscitation in critically ill patients, long-term chemotherapy in tumor patients, parenteral nutrition support in postoperative surgical patients, and blood purification in patients with chronic renal failure, venous catheters are used in hundreds of millions worldwide each year. However, the incidence of venous catheter-related complications is as high as 20%−30%, and FS, as the earliest-formed biofilm structure, has become the primary pathological basis of catheter dysfunction [8–12]. Studies have shown that FS can start to form within 24 hours after catheterization, and it causes clinical harm mainly through the following mechanisms: mechanical obstruction: sheath thickening reduces the effective inner diameter of the catheter, leading to increased infusion resistance; thrombogenicity: the fibrin network promotes platelet adhesion, increasing the risk of catheter-related thrombosis; vascular wall injury: persistent venous inflammatory response induces venous endothelial edema and fibrotic remodeling, accelerating vascular dysfunction [13–14]. It is worth noting that current clinical interventions for FS are mostly passive treatments based on the appearance of symptoms (such as urokinase thrombolysis and antiplatelet therapy), but there is still a gap in the understanding of the ultra-early dynamic evolution of FS and its vascular injury mechanism [15–20]. At present, studies on the impact of FS on blood vessels mostly focus on a single time point, lacking systematic research on its ultra-early dynamic change process. In particular, the time-sequential variation patterns of the impact of FS on blood vessels within hours to days after catheterization are still unclear, such as how the infusion patency gradually decreases over time, what changes the thickness and structural integrity of FS show at different stages, how the thrombus area and inflammatory response evolve over time, and what is the correlation between the degree of vascular edema and FS formation, which still need to be further explored.

This study established an animal experimental model of venous indwelling catheter-related FS based on the rabbit ear marginal vein, selected multiple representative ultra-early time nodes such as 12h, 1d, 2d, 3d, and 4d of indwelling for sampling, and used the classic Hematoxylin-Eosin (HE) staining technique to visually observe the pathological changes of blood vessels at the histological level. Taking infusion patency, FS thickness, FS continuity, thrombus area, inflammatory cell infiltration, and venous edema as the core observation indicators, the time-sequential variation patterns of the impact of venous catheter-related FS on blood vessels were systematically explored. The development of this study, on the one hand, helps to deeply reveal the dynamic relationship between FS formation and vascular injury, and enriches the basic theoretical research on venous catheter-related complications; on the other hand, by clarifying the key time nodes and pathological characteristics of the impact of FS on blood vessels, it is expected to provide a theoretical basis for clinical formulation of more scientific and reasonable venous catheter management strategies, such as optimizing catheter replacement time and early intervention in FS formation, so as to reduce the incidence of catheter-related complications and improve the clinical treatment effect and patient prognosis quality.

## Materials and methods

### Experimental animals

Forty healthy adult New Zealand white rabbits (20 males and 20 females), weighing 2.0–2.5 kg, were provided by the Animal Experiment Center of Guilin Medical University. All animals were housed in an SPF-grade animal facility complying with national standards, with the environmental temperature controlled at 22 ± 2°C, relative humidity maintained at 50%–60%, a 12-h light/dark cycle, and free access to food and water. They were acclimatized for 7 days before the experiment to minimize the impact of environmental changes on the results. During the feeding period, the rabbits’ mental state, food intake, and fecal condition were observed daily to ensure no health abnormalities. This experiment was approved by the Animal Ethics Committee of Guilin Medical University (Approval No.: GLMC202511024), and the procedures strictly followed the principles of animal welfare and ethics.

### Experimental materials

#### Instruments.

The instruments included a thermostatic surgical table (to maintain animal body temperature during surgery), a small-animal-specific respiratory anesthesia machine, a microsurgical instrument set (including fine hemostatic forceps, microsurgical needle holders, ophthalmic scissors, forceps, etc.), a low-speed centrifuge, a paraffin microtome, a microscope imaging system (equipped with image analysis software for quantitative analysis), an infusion pump (accuracy ±0.1 ml/h), an electronic balance (accuracy 0.001 g), a vernier caliper (accuracy 0.02 mm), etc. All instruments were calibrated and tested for performance before use to ensure the accuracy and reliability of experimental data.

#### Reagents.

Heparin sodium injection (12,500 U/vial) was used for anticoagulation, prepared as 10 U/ml heparin saline.4% paraformaldehyde solution was used for tissue fixation.Gradient alcohols (70%, 80%, 90%, 95%, 100%) and xylene were used for tissue dehydration and clearing.Paraffin was used for tissue embedding. Hematoxylin-eosin (HE) staining kit.Neutral gum was used for coverslipping.Normal saline was used for intraoperative flushing and postoperative maintenance of the infusion pathway.All reagents were purchased from qualified biological reagent companies and stored/used strictly according to the instructions.

#### Consumables.

The catheters used in this study were 24-gauge intravenous catheters made of medical-grade polyurethane (Haifeng Venous Indwelling Needle, model: Y-type 24G), made of medical-grade polyurethane with a smooth surface and good biocompatibility,This material is widely used in clinical settings due to its biocompatibility and flexibility.Disposable medical consumables including sterile surgical dressings, 5−0 absorbable sutures, sterile gloves, syringes, etc., to ensure aseptic operation.

#### Grouping design.

Forty New Zealand white rabbits were randomly divided into 5 groups (8 rabbits per group): 12-h indwelling group, 1-d indwelling group, 2-d indwelling group, 3-d indwelling group, and 4-d indwelling group. Grouping was performed using a random number table to ensure no significant differences in gender, body weight, etc., between groups (P > 0.05).

#### Model establishment procedure.

Rabbits were fasted for 12 h before the experiment but had free access to water.Rabbits were placed in an induction anesthesia chamber and induced with 3% isoflurane inhalation. After the corneal reflex weakened and muscles relaxed, they were fixed supine on a thermostatic surgical table, connected to a respiratory anesthesia machine, maintained at anesthetic depth of 1.5%–2% isoflurane, and vital signs (heart rate, respiratory rate, oxygen saturation, etc.) were monitored.The marginal ear vein of the rabbit was selected as the catheterization vessel. The ear skin was disinfected three times with povidone-iodine (5 cm diameter) and draped with a sterile surgical towel.A 24G indwelling venous needle was slowly inserted into the vein. After confirming unobstructed blood return, the needle core was removed, and the indwelling catheter was advanced 2–3 cm along the vessel direction to ensure stability within the blood vessel.The wound was disinfected again with povidone-iodine, applied with an appropriate amount of antibiotic ointment to prevent infection, and the indwelling needle was fixed to the skin surface with a 3M dressing to prevent catheter displacement or dislodgement.After successful catheterization, 10 U/mL heparin saline was used for catheter locking. During the subsequent indwelling period, to observe the natural formation process of the fibrin sheath, no routine flushing and maintenance of the catheter were performed. Only before sample collection at the predetermined time points for each group, an infusion pump was connected to infuse normal saline at a rate of 10 mL/h to test the patency of the catheter.After surgery, rabbits were returned to their cages, housed individually, and closely observed for recovery and postoperative status.Success criteria of the model:① Macroscopic observation: The surface of the extracted catheter is covered with milky white translucent membranous substances;② Fix the venous segment containing the catheter with 4% paraformaldehyde, and perform paraffin sectioning. HE staining shows the organizational structure of the fibrin sheath.

#### Sample collection.

**Fig 1:**
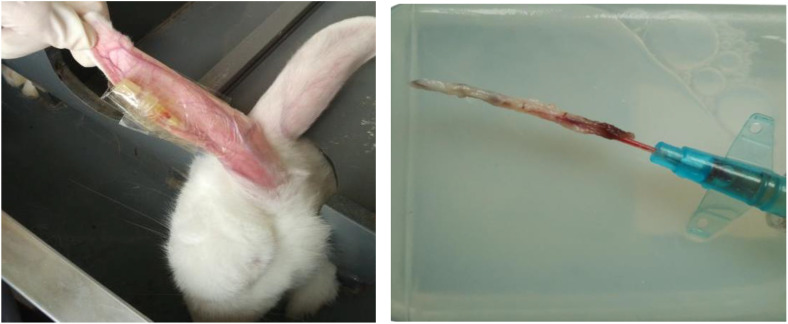
FS sample.

Rabbits in each group were euthanized and sampled according to the set indwelling times. Before euthanasia, venous infusion patency was detected: an infusion pump was connected to the indwelling catheter, and normal saline was infused at a set speed of 10 ml/h. Resistance during infusion and stability of the infusion speed were observed, and patency (patent, partially blocked, completely blocked) was recorded. Euthanasia was performed by intraperitoneal injection of excessive pentobarbital sodium (80–100 mg/kg). The skin was incised along the course of the marginal ear vein, and a venous segment (3–4 cm long) containing the indwelling catheter and surrounding tissue was completely isolated. The obtained venous tissue was immediately fixed in 4% paraformaldehyde solution for 24 h, then rinsed three times with PBS buffer (10 min each) to remove residual fixative (Fig 1).

#### HE staining.

The fixed venous tissues were sequentially dehydrated in gradient alcohols (70%, 80%, 90%, 95%, 100%, 1 hour each), then cleared twice in xylene for 30 minutes each. The cleared tissues were embedded in melted paraffin, and 4-μm-thick sections were cut using a paraffin microtome. Sections were dewaxed twice in xylene (5 minutes each), rehydrated through gradient alcohols (100%, 95%, 90%, 80%, 70%, 3 minutes each), stained with hematoxylin for 5 minutes, rinsed with tap water for 1 minute, differentiated with 1% hydrochloric acid alcohol for 3–5 seconds, and blued in tap water for 5 minutes. Eosin staining followed for 3 minutes, then dehydration through gradient alcohols (70%, 80%, 90%, 95%, 100%, 3 minutes each), twice clearing in xylene (5 minutes each), and coverslipping with neutral gum.

### Observation indicators

#### FS thickness measurement.

Under a microscope, the thickest FS region on each section was selected, and FS thickness was measured in 5 random fields using the scale function of the microscope imaging system (unit: μm). The average value was taken as the FS thickness of the sample.

#### FS structural integrity assessment.

Two pathologists with rich experience observed sections using a double-blind method, classifying structural integrity into three grades: complete, mostly complete, and incomplete.Complete: FS continuity reaches 100%.Mostly complete: FS continuity exceeds 75%.Incomplete: FS continuity is less than 50%.

#### Thrombus area calculation.

Image analysis software processed section images captured under a microscope. Thrombus regions were segmented by setting appropriate thresholds, and the software automatically calculated the thrombus area (unit: mm²). Three random fields were measured per sample, with the average as the thrombus area.

#### Inflammatory cell infiltration assessment.

Under high power (×400), 5 random fields were selected to count inflammatory cells (mainly neutrophils, lymphocytes, etc.), with the average as the quantitative index. Inflammation was graded as:Mild: Scattered inflammatory cells in perivascular tissues.Moderate: Focal or patchy distribution with wide infiltration.Severe: Dense infiltration with tissue necrosis and other severe lesions.

#### Venous edema assessment.

Venous wall morphology was observed, and wall thickness was measured by the inner-outer diameter difference in 5 random fields (average value). Edema was graded as:No edema: Clear wall structure, no thickness change.Mild edema: Slight thickening, basically clear structure.

Moderate edema: Obvious thickening, blurred structure.Severe edema: Significant thickening with widened tissue spaces and cell degeneration/necrosis.

### Data analysis

SPSS 26.0 was used. Continuous variables (FS thickness, thrombus area, inflammatory cell count) with normal distribution (Shapiro-Wilk test) were expressed as *x* ± *s*. Due to unequal variances (Levene test, P < 0.05), Welch’s ANOVA with Games-Howell post hoc test was used for intergroup comparison. Categorical variables (patency, sheath continuity, inflammation/edema degree) were expressed as frequency (%), and Fisher-Freeman-Halton exact test was used (>20% cells with expected frequency <5). Significance level: α = 0.05 (two-sided).

## Results

### Infusion patency

Patency significantly decreased with indwelling time (Cochran-Armitage trend test, p < 0.001). The patency rate dropped from 87.5% at 12 hours to 0% at 4 days, with complete blockage increasing to 75.0%. Intergroup distribution differed significantly (Fisher-Freeman-Halton exact test, p < 0.001), and indwelling time showed a strong negative correlation with patency (Spearman’s ρ = −0.782, p < 0.001) (see Table 1).

**Table 1:** Comparison of infusion patency at different indwelling times (n, %).

Indwelling Time	Sample Size	Patent (n)	Partially Blocked (n)	Completely Blocked (n)	Patency Rate (%)
12h	8	7	1	0	87.5
1d	8	5	2	1	62.5
2d	8	3	3	2	37.5
3d	8	1	3	4	12.5
4d	8	0	2	6	0

### FS thickness

**Fig 2:**
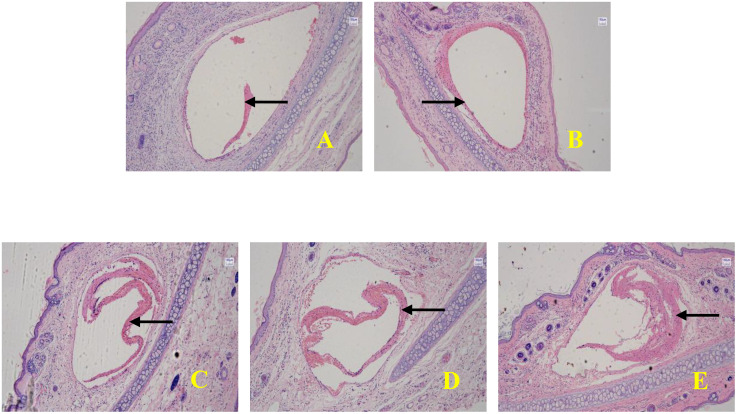
Trend diagram of fibrin sheath thickness at different indwelling times (HE staining, × 10) (Note: A: Indwelling for 12 h; B: Indwelling for 1 d; C: Indwelling for 2 d; D: Indwelling for 3 d; E: Indwelling for 4 d.The black arrows indicate the location of FS.).

The thickness of FS increased extremely significantly with the prolongation of indwelling time (Welch’s ANOVA, F = 625.4, p < 0.001), and the growth trend showed a strong linear relationship (slope b = 33.3 μm/time unit, p < 0.001). The thickness increased from 23.5 ± 4.2 μm at 12 h to 156.7 ± 10.8 μm at 4 d, and the differences at all adjacent time points were significant (Games-Howell test, p < 0.001). The indwelling time explained 98.6% of the thickness variation (η² = 0.986), confirming it as a key predictor. (see Table 2 and Fig 2).

**Table 2:** Comparison of FS thickness at different indwelling times (x ± s, μm).

Indwelling Time	Sample Size	FS thickness	95%CI
12h	8	23.5 ± 4.2	19.9-27.1
1d	8	45.8 ± 6.3	40.7-50.9
2d	8	78.2 ± 8.1	72.1-84.3
3d	8	112.3 ± 9.5	105.1-119.5
4d	8	156.7 ± 10.8	148.1-165.3

### FS structural integrity

**Fig 3:**
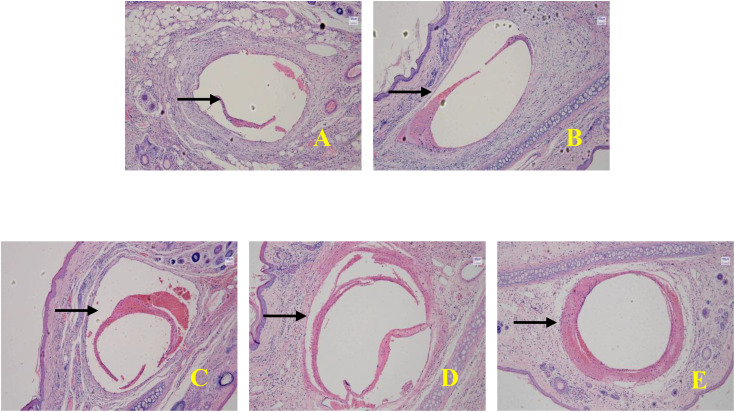
Structural integrity of FS at different indwelling times (HE staining, × 10) (Note: A: Indwelling for 12 h; B: Indwelling for 1 d; C: Indwelling for 2 d; D: Indwelling for 3 d; E: Indwelling for 4 d.The black arrows indicate the location of FS.).

Indwelling time had a significant impact on FS structural integrity, demonstrating a strong positive trend (Jonckheere-Terpstra trend test, J = 580, p < 0.001). As indwelling time increased from 12 hours to 4 days, the integrity grade gradually improved:In the early stage (≤1 day), it was predominantly incomplete (incomplete proportion ≥75.0%, no complete samples).In the middle stage (2–3 days), it transitioned to mostly complete and complete (e.g., the complete proportion increased to 25.0% at 2 days and reached 62.5% at 3 days).In the late stage (4 days), it was predominantly complete (complete proportion 87.5%, no incomplete samples).The intergroup distribution differed significantly (Fisher-Freeman-Halton exact test, p < 0.001). The average integrity score increased from 1.125 at 12 hours to 2.875 at 4 days (Spearman’s ρ = 0.782, p < 0.001), confirming that prolonged indwelling time is a strong predictor of improved integrity (see Table 3 and Fig 3).

**Table 3:** Comparison of FS structural integrity at different indwelling times (n, %).

Indwelling Time	Sample Size	Incomplete	Mostly complete	Complete
12h	8	7 (87.5)	1 (12.5)	0 (0)
1d	8	6 (75.0)	2 (25.0)	0 (0)
2d	8	1 (12.5)	5 (62.5)	2 (25.0)
3d	8	1 (12.5)	2 (25.0)	5 (62.5)
4d	8	0 (0)	1 (12.5)	7 (87.5)

### Thrombus area

The thrombus area increased extremely significantly with the prolongation of indwelling time (Welch’s ANOVA, F = 498.6, p < 0.001), and the growth trend showed a strong linear relationship (slope b = 0.435 mm²/time unit, p < 0.001). The area increased from 0.12 ± 0.03 mm² at 12 h to 1.86 ± 0.20 mm² at 4 d, and the differences at all adjacent time points were significant (Games-Howell test, p < 0.001). Indwelling time explained 97.6% of the area variation (η² = 0.976), making it a key determinant (see Table 4).

**Table 4:** Comparison of thrombus area at different indwelling times (x ± s, mm²).

Indwelling Time	Sample Size	Thrombus area	95%CI
12h	8	0.12 ± 0.03	0.094-0.146
1d	8	0.35 ± 0.07	0.292-0.408
2d	8	0.78 ± 0.12	0.682-0.878
3d	8	1.25 ± 0.15	1.128-1.372
4d	8	1.86 ± 0.20	1.702-2.018

### Inflammatory cell infiltration

Inflammatory response significantly intensified with indwelling time: the number of inflammatory cells (primarily neutrophils and lymphocytes) increased from 12.3 ± 2.5 cells/field at 12 h to 85.4 ± 6.3 cells/field at 4 d (Welch’s ANOVA, p < 0.001), and the proportion of severe inflammation rose from 0% to 62.5% (Cochran-Armitage trend test, p < 0.001). Inflammatory cell counts differed significantly between all adjacent time points (Games-Howell, p < 0.001), and indwelling time showed a strong correlation with inflammation severity (Spearman’s ρ = 0.89, p < 0.001). Intervention within 72 hours is recommended to avoid the risk of severe inflammation (see Table 5).

**Table 5:** Comparison of inflammatory cell infiltration at different indwelling times.

Indwelling Time	Sample Size	Number of inflammatory cells (*x* ± *s*, cells/field)	Mild inflammation (n，%)	Moderate inflammation (n，%)	Severe inflammation (n，%)
12h	8	12.3 ± 2.5	6 (75.0)	2 (25.0)	0 (0)
1d	8	28.6 ± 3.8	4 (50.0)	4 (50.0)	0 (0)
2d	8	45.7 ± 4.6	2 (25.0)	6 (75.0)	0 (0)
3d	8	62.1 ± 5.2	1 (12.5)	4 (50.0)	3 (37.5)
4d	8	85.4 ± 6.3	1 (12.5)	2 (25.0)	5 (62.5)

### Degree of venous edema

The degree of venous edema significantly deteriorated with the prolongation of indwelling time (Jonckheere-Terpstra trend test, p < 0.001). In the early stage (12 h), mild edema was predominant (62.5%), while severe edema dominated at 4 days of indwelling (62.5%). The proportion of moderate to severe edema increased from 0% to 75.0% (Cochran-Armitage trend test, p < 0.001), with significant differences in intergroup distribution (Fisher-Freeman-Halton test, p < 0.001). Indwelling time was strongly correlated with the degree of edema (Spearman’s ρ = 0.73, p < 0.001). Intervention within 72 hours is recommended to avoid the risk of severe edema (see Table 6).

**Table 6:** Comparison of venous edema at different indwelling times (n, %).

Indwelling Time	Sample Size	No edema	Mild edema	Moderate edema	Severe edema
12h	8	3 (37.5)	5 (62.5)	0 (0)	0 (0)
1d	8	2 (25.0)	3 (37.5)	3 (37.5)	0 (0)
2d	8	1 (12.5)	3 (37.5)	4 (50.0)	0 (0)
3d	8	0 (0)	3 (37.5)	3 (37.5)	2 (25.0)
4d	8	0 (0)	2 (25.0)	1 (12.5)	5 (62.5)

## Discussion

This study systematically explored the time-series changes in the effects of fibrin sheath (FS) on blood vessels at different ultra-early indwelling times by establishing an animal model of FS related to indwelling catheters in the auricular marginal veins of rabbits. The results not only provide a new perspective for understanding the pathogenesis of venous catheter-related complications but also have important guiding significance for optimizing clinical venous catheter management strategies, which will be discussed in depth from multiple aspects below.

### Physiological and pathological mechanisms of FS formation and development

This study confirms that FS formation is rapidly initiated after venous catheterization, and its thickness shows a significant linear increase with indwelling time. This process is closely related to the body’s defensive physiological response to foreign bodies. When a venous catheter is implanted as a foreign body into a blood vessel, components such as blood proteins, platelets, and fibrinogen deposit on the catheter surface, triggering a complex coagulation cascade. FS begins to take shape 12 hours after catheterization. As time progresses, continuous activation of coagulation factors and cross-linking polymerization of fibrin lead to gradual thickening of the sheath. This continuous growth trend is consistent with previous theories about biofilm formation, further revealing the key role of FS as the earliest-formed biofilm structure in the occurrence of venous catheter-related complications.

This study also found that the retention time is a key factor affecting the structural integrity of FS, and its positive promoting effect is statistically significant (p < 0.001). As the retention time extends from 12 hours to 4 days, the integrity shows a clear stage-by-stage evolution pattern. This phenomenon may be related to the biomechanical remodeling or molecular assembly dynamics of FS. The incomplete structure in the early stage (≤1 day) may be related to insufficient matrix component deposition or incomplete tissue cross-linking, while the rapid improvement of integrity in the middle stage (2–3 days) may reflect the progressive assembly and structural reinforcement of the extracellular matrix. The high integrity ratio at the 4-day retention period suggests that this time point may approach the critical threshold for FS structural stability. The significance of the difference in intergroup distribution (p < 0.001) further supports the biological significance of retention time as an independent predictor. It is worth noting that the results of this study provide a quantitative basis for the retention strategy of FS-related samples, but more biological indicators still need to be combined to explore the specific mechanisms of structural integrity improvement, such as matrix protein expression or changes in cell activity.

### Multidimensional effects of FS on vascular function

Infusion Patency and Mechanical Obstruction The results show that the infusion patency rate significantly decreases with prolonged indwelling time, with a deterioration inflection point at 3–4 days of indwelling, which is consistent with the clinical phenomenon of poor infusion after prolonged use of venous indwelling catheters [21,22]. In the early stage of indwelling (12 hours), the infusion patency rate is high because FS is still in the initial formation stage, causing minimal blockage to the catheter lumen. However, as time goes on, FS continues to thicken, and its internal structure becomes denser, increasing compression and obstruction of the lumen, leading to higher infusion resistance and a significant decrease in patency rate. This indicates that FS thickening is a key factor affecting infusion patency and provides an experimental basis for clinical timely catheter replacement to prevent infusion complications. In clinical practice, this phenomenon may lead to slowed drug infusion, inaccurate dosage, or even complete catheter blockage, affecting treatment efficacy. Therefore, accurately grasping the time node and development law of FS-induced infusion obstruction is of great significance for clinical timely intervention.

Thrombosis Risk and Thrombogenic Mechanism This study confirms that the thrombus area shows a significant linear increase with indwelling time, explaining the clinical phenomenon of gradually increasing thrombus complications related to venous indwelling catheters and further confirming the thrombogenic effect of FS. The rough surface of FS provides an ideal site for platelet adhesion and aggregation, while the internal fibrin network structure also facilitates the attachment and deposition of blood cells, promoting thrombus formation and development [23]. As FS thickens and its continuity is disrupted, its stimulation to vascular endothelium increases, leading to vascular endothelial cell injury and further activation of the coagulation system, which also promotes thrombus formation [24,25]. Once formed, thrombi can not only block the catheter but also detach into the bloodstream, causing serious complications such as pulmonary embolism and cerebral embolism, threatening the patient’s life. In hemodialysis treatment for patients with chronic renal failure, catheter-related thrombosis is one of the common and serious complications. The results of this study help to deeply understand its pathogenesis, provide a basis for formulating more effective anticoagulant treatment plans and thrombosis prevention strategies in clinical practice, and emphasize the importance of early thrombosis prevention.

Inflammatory Response and Vascular Wall Injury The results that the degree of inflammatory cell infiltration and venous edema gradually worsen with indwelling time indicate that FS formation triggers a strong inflammatory response in the body, thereby causing vascular wall injury. When FS is formed in the blood vessel, it activates the body’s immune system, attracting inflammatory cells such as neutrophils and lymphocytes to gather around the blood vessel. Inflammatory mediators released by these inflammatory cells, such as tumor necrosis factor-α (TNF-α) and interleukin-6 (IL-6), can damage vascular endothelial cells, increase their permeability, lead to fluid exudation from blood vessels, and cause venous edema [26,27]. Meanwhile, persistent inflammatory response can also induce edema and fibrotic remodeling of venous endothelial cells, accelerate vascular dysfunction, and ultimately affect the service life of venous catheters and the patient’s treatment effect.

As indwelling time increases, the body’s inflammatory response gradually intensifies. Various cytokines and chemokines released by inflammatory cells, such as platelet-derived growth factor and transforming growth factor-β, can promote the proliferation and migration of fibroblasts and accelerate fibrin deposition, leading to a rapid increase in FS thickness [28–30]. This change not only affects catheter function but also may increase the risk of thrombus formation and detachment. With prolonged indwelling time, the inflammatory response continues to intensify, and the damage to blood vessels becomes more severe, which provides a new perspective for understanding the pathological mechanism of venous indwelling catheter-related vascular complications.

### Guiding significance of research results for clinical practice

Optimizing Catheter Replacement Time Based on the changes in infusion patency, FS thickness, and related complications over time in this study, important references can be provided for clinical optimization of venous catheter replacement time. The results show that indwelling time exceeding 48 hours enters the critical point of the vascular injury cascade reaction (thrombus area > 0.75 mm², sheath thickness > 78 μm, moderate to severe inflammation/edema > 75%), while exceeding 72 hours reaches the high-risk point (complete blockage rate > 50%, severe edema > 25%, complete sheath fracture > 50%). Therefore, it is recommended that for patients with long-term indwelling catheters, intensive evaluation should be conducted during 48–72 hours of indwelling, and catheters should be replaced in a timely manner when necessary based on comprehensive judgments of catheter patency, patient coagulation, and inflammatory status to reduce the risk of complications. This strategy helps improve the safety and effectiveness of infusion therapy and reduce treatment interruptions and adverse events caused by catheter dysfunction.

Formulating Early Intervention Strategies Clarifying the ultra-early dynamic evolution law of FS provides a theoretical basis for formulating clinical early intervention strategies. Since FS begins to form and develop shortly after catheterization, early intervention is particularly important. At present, clinical interventions for FS are mostly passive. However, the results of this study suggest that measures can be taken in the early stage after catheterization, such as using anticoagulant drugs to inhibit the coagulation cascade and anti-inflammatory drugs to reduce the inflammatory response, to delay the formation and development of FS. In addition, the research and development of new materials and technologies, such as venous catheters with anticoagulant and anti-adhesion properties on the surface, are also expected to reduce FS formation and complication rates from the source.

Individualized Catheter Management Different patients have different tolerances and responses to venous catheters, and the results of this study provide a basis for implementing individualized catheter management. Clinicians can formulate personalized catheter management plans according to the patient’s condition, underlying diseases, coagulation function, and other factors, combined with the time-series changes in the effects of FS on blood vessels in this study. For example, for patients with hypercoagulability or high coagulation status, the catheter indwelling time can be appropriately shortened, and anticoagulant therapy can be strengthened; for patients with a strong inflammatory response, anti-inflammatory drug intervention can be given to reduce the risk of FS-related complications.

## Research limitations and future prospects

Although this study has achieved certain results, there are still some limitations. First, only New Zealand white rabbits were used as experimental animals. Although the auricular marginal veins of rabbits have some similarities with human veins in anatomical structure and physiological function, they cannot fully simulate the complex human physiological environment and pathological state, which may lead to certain deviations when extrapolating the research results to humans. Future studies can further optimize animal models or carry out clinical studies to more accurately reveal the formation mechanism of FS in humans and its effects on blood vessels.

Second, this study only observed the pathological changes of FS and blood vessels through HE staining, without combining other detection methods, such as immunohistochemical staining and electron microscopy observation, to further explore the mechanism at the molecular and ultrastructural levels. Additionally, this study only selected limited time points such as 12 hours, 1 day, 2 days, 3 days, and 4 days of indwelling for observation, and the impact of FS on blood vessels over a longer time range has not been deeply explored. In clinical practice, many patients’ venous catheters need to be indwelling for weeks or even months. Therefore, future studies should extend the observation time to investigate the change law of FS and its chronic effects on blood vessels under longer indwelling conditions.

Furthermore, this study mainly observed the pathological changes of blood vessels at the histological level, and the research on the molecular mechanism and dynamic changes of hematological indicators during FS formation is insufficient. In-depth exploration of the key molecules and signaling pathways involved in FS formation, such as the mechanisms of action of coagulation factors, inflammatory mediators, and cell adhesion molecules, will help reveal the essence of FS formation and vascular injury. At the same time, combining dynamic monitoring of hematological indicators, such as coagulation function indicators and inflammatory factor levels, can more comprehensively evaluate the impact of FS formation on the body and provide more accurate diagnostic and treatment bases for clinical practice.

Lastly, this study did not systematically measure and calculate the catheter-to-vein ratio (CVR). A high CVR (i.e., a relatively large-bore catheter) is known to exacerbate mechanical injury to the vascular endothelium and alter hemodynamics, which may potentially accelerate fibrin sheath and thrombus formation. Although we attempted to control for variability in CVR by standardizing animal weight, catheter model, and insertion site, the failure to quantify it as an independent variable remains a limitation. Future studies could be specifically designed to investigate the impact of different CVRs on the chronology and severity of FS formation, thereby providing more precise experimental evidence for clinical selection of the most appropriately sized catheters.

## Conclusion

In conclusion, this study confirms through animal experiments that venous catheter-related FS has obvious time-series effects on blood vessels, and the injury to blood vessels gradually worsens with prolonged indwelling time. This result provides important experimental evidence for deeply understanding the formation mechanism of FS and the occurrence and development of related complications, and also provides theoretical support for clinical optimization of the use scheme of venous indwelling catheters, such as reasonable selection of catheterization time, strengthening catheter maintenance, and early prevention of complications. Future studies can further expand the types of experimental animals, combine multiple detection technologies, and explore the interaction mechanism between FS and blood vessels from a more comprehensive and in-depth perspective, so as to provide more effective strategies for the clinical prevention and treatment of venous indwelling catheter-related complications.
